# Hierarchical Graphene/Au/Polyaniline Nanostructured Electrode for Dual-Modality Electrochemical LAMP Biosensing of *Helicobacter pylori*

**DOI:** 10.1021/acs.analchem.5c04200

**Published:** 2025-11-04

**Authors:** Rajkumar Rakesh Kumar, Chi-Yu Wang, Aditya Manu Bharti, Wen-Chian Hu, Yi-Chen Zhang, Ling-Shan Yu, Pornnapat Tungtrakarnkul, Kevin C.-W. Wu, Cheng-Hsin Chuang

**Affiliations:** † Institute of Medical Science and Technology, 34874 National Sun Yat-Sen University, Kaohsiung 80424, Taiwan; ‡ Institute of Biomedical Engineering and Nanomedicine, National Heath Research Institutes, Miaoli 350401, Taiwan; § Medical Cosmetic Institution, 63430 Kaohsiung Armed Forces General Hospital, Kaohsiung 802301, Taiwan; ∥ Institute of Biopharmaceutical Science, National Sun Yat-Sen University, Kaohsiung 80424, Taiwan; ⊥ Department of Biomedical Engineering, 26685 Mahidol University, Nakhon Pathom 73170, Thailand; # International PhD Program for Science, National Sun Yat-Sen University, Kaohsiung 80424, Taiwan; ¶ Department of Chemical Engineering, National Taiwan University, No. 1, Sec. 4, Roosevelt Road, Taipei 10617, Taiwan; ▽ Department of Chemical Engineering and Materials Science, Yuan Ze University, Chung-Li, Taoyuan 32003, Taiwan

## Abstract

*Helicobacter pylori* (HP) infection is directly associated with over 90% of all gastric cancer (GC) cases. Currently available HP tests are prone to false negatives and are inapt for decentralization. Reliable and user-friendly detection platforms for timely diagnosis and routine monitoring are critical to patient survival. Herein, we targeted this unmet need by developing a novel biosensing platform that strategically combined the robust versatility of electrochemical techniques with the sensitivity and specificity of loop-mediated isothermal amplification (LAMP) to enable the accurate detection of HP at the point-of-care (POC). The biosensor design consisted of an underlying screen-printed carbon electrode (SCPE), sequentially modified with (i) highly conductive acid-functionalized sp2 graphene (Gr) and (ii) needle-like gold microstructures encapsulated with (iii) pH-sensitive polyaniline (PANI), assembled between laser-cut cover layers with a built-in reaction chamber for facile sample handling and detection. LAMP was performed using synthesized primers targeting the HP glmM gene. The amplification inherently generates H^+^, triggering pH variations, which are precisely tracked by the developed biosensor to simultaneously monitor amplicon growth and quantify HP DNA concentrations via two modes: continuous mode and segmented mode. High sensitivity in a wide linear range (1 to 107 copies/μL) with a limit of detection (LOD) of 1 copy/μL is reported. Furthermore, the excellent correlation with clinical results underscored the practical feasibility of this platform to allow reliable early diagnosis of HP infections and serve as a viable alternative to gastric endoscopy.

## Graphical abstract

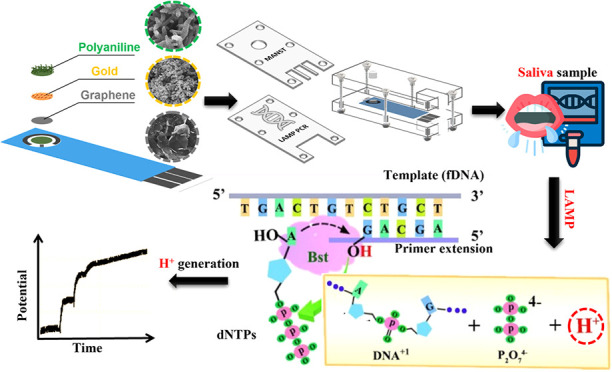

## Introduction

Gastric cancer (GC) is the fifth most common form of malignancy, known for its elevated end-stage mortality and consistently poor prognosis. Approximately 1 in 5 patients survives more than 5 years postdiagnosis.^1^ The asymptomatic nature of the early stages further contributes to delayed diagnosis, leading to unnoticed proliferation and lack of timely medical interventions. Thus, GC is established as the fourth leading cause of all cancer-related mortality. The 2020 Global Cancer Observatory (GLOBOCAN) report estimated more than a million cases of GC worldwide, which is expected to increase by 62% to 1.77 million by 2040.^2^ Although several risk variables for GC, including dietary factors,^3^ high body fat percentage,^1^ smoking^4^ and conditions such as Epstein–Barr virus infection,^5^ autoimmune gastritis,^6^ Ménétrier disease^7^ etc. have been recognized, *Helicobacter pylori* (HP) infections remain the primary etiological factor.^8^

HP is a Gram-negative bacterium classified as a class I human carcinogen with a global burden of more than 4.3 billion positive cases.^9^ secretes the enzyme urease, which can decompose small amounts of urea in the stomach into ammonia and carbon dioxide to neutralize gastric acid, allowing HP to persist in gastric mucus, gastric mucosal cells, and the duodenum for extended periods.^10^ Furthermore, the absence of characteristic symptoms often confers false diagnoses and ineffective treatments. Consequently, the resulting perpetual complications can cause severe consequences, including gastric cancer (GC).^11^ Based on epidemiological evidence, the International Agency for Research on Cancer (IARC) has reconfirmed HP infection as a ’necessary but insufficient” cause for GC,^12^ noting that almost 90% of all GC were associated with HP infection. Therefore, Therefore, a fast and accurate diagnosis is crucial to improve patient prognosis a fast and accurate diagnosis is crucial to improve patient prognosis. Currently available clinical tools for *H. pyroli* HP diagnostics, such as gastric endoscopy,^13^ allow direct symptom evaluation while allowing subsequent analysis, including rapid urease tests^14^ and histological examinations.^15^ However, its invasive nature can cause discomfort, leading to patient reluctance.^16^ Meanwhile, serum antibody tests are restricted by the characteristic persistence of antibodies even after infection clearance, thus limiting the real-time infection assessment.^17^

Alternatively, noninvasive screening methods such as the 13C breath test demonstrate HP detection indirectly via urease activity.^18^ However, the presence of other urease-producing bacteria can lead to false positives, and the utilization of isotopes necessitates specialized instruments, rendering this test highly costly.^19^ Meanwhile, fecal antigen detection is prone to false negatives due to stool sample variability.^20^ To this end, polymerase chain reaction (PCR) serves as an ideal alternative, offering high specificity and minimal false positives, while enabling candidate with substantial advantages, including high specificity for minimal false positives to enable quantitative detection of HP infections.^21^ However, currently available commercial PCR kits for HP detection often involve invasive forms of sample collection in addition to expensive thermocyclers with exhaustive temperature controls,^22^ thus limiting their decentralized application in much-needed resource-constrained settings, particularly in developing countries, where HP infection rate are highest.^23^

Loop-mediated isothermal amplification (LAMP) can overcome these limitations of traditional PCR. Its innate specificity, high efficiency, and capabilities for rapid isothermal amplification under 65 °C make it particularly suitable for personalized and point-of-care (POC) testing. Furthermore, electrochemical biosensing platforms offer significant advantages for decentralized medicine owing to their sensitivity,^24^ reliability,^25^ cost-effectiveness,^26^ and simple detection mechanism,^27^ which allows for easy miniaturization^28^ and integration with numerous versatile protocols, including LAMP. As such, electrochemical biosensors are widely implemented in various research fields, including clinical,^29,30^ environmental,^31^ personalized medicine,^32^ industrial,^33^ and agricultural^34^ applications.

Existing HP detection technologies span a wide range of sensing strategies but remain limited in their practicality and analytical robustness. Impedimetric platforms employing antibody-assisted capture of protein biomarkers such as HopQ have shown promise in controlled environments like water samples.^35^ However, they rely on multiple washing and incubation steps, making them unsuitable for direct use in complex biological matrices. Colorimetric biosensors offer rapid HP detection but suffer from semiquantitative outputs and poor analytical resolution.^36^ Similarly, fluorescence-based assays coupled with CRISPR-Cas12a integrated hairpin-mediated self-primer exponential amplification (HSEA)^37^ or loop-mediated isothermal amplification (LAMP)^38^ provide high sensitivity but require invasive sample collection and labor-intensive purification, hindering point-of-care implementation.

This work addresses the critical need for efficient, noninvasive detection of HP in complex biological samples by integrating the high specificity of loop-mediated isothermal amplification (LAMP) with the user-friendly transduction of electrochemical techniques. A dual-mode electrochemical LAMP biosensing platform was developed based on a hierarchical graphene–gold–polyaniline (Gr/Au/PANI) electrode architecture that directly converts LAMP-induced pH variations into quantifiable electrochemical signals without mediators or labeling, enabling accurate real-time monitoring of HP DNA amplification in saliva samples. A strategic segmented detection mode was introduced in an electrochemical LAMP system to minimize signal drift from prolonged reagent–electrode interactions, significantly enhancing the sensitivity and reproducibility. The in-house-fabricated screen-printed carbon electrode (SCPE) with a three-electrode configuration was sequentially modified with acid-functionalized sp^2^ graphene, dendritic gold microstructures, and pH-sensitive polyaniline under optimized conditions to form a highly electroactive multilayer biochip. Comprehensive physicochemical (FTIR, EDS, contact angle), morphological (HR-TEM, FESEM), and electrochemical (CV, EIS with circuit modeling) analyses validated the electrode architecture. The optimized biosensor enabled dual-mode detection (continuous and segmented) to monitor LAMP amplicon growth and quantify HP DNA concentrations, offering a portable, low-cost, and high-fidelity solution for decentralized HP diagnostics.

## Materials and Methods

### Materials

Hydrogen tetrachloroaurate trihydrate (III) (HAuCl_4_, 99.99%), hydrochloric acid (HCl, 37%), sulfuric acid (H_2_SO_4_, 95.0–98.0%), aniline (C_6_H_5_NH_2,_ 99.5%), potassium hexacyanoferrate (III) (K3Fe (CN) 6, 99.0%), potassium hexacyanoferrate trihydrate (II) (K_4_Fe (CN)_6_·3H_2_O, 98.5–102.0%), and artificial saliva were purchased from Sigma-Aldrich. Sodium hydroxide pellets (NaOH, 97%) were purchased from SHOWA Chemical Co., Ltd. Phosphate-buffered saline (PBS) was acquired from UniRegion Bio-Tech. All solutions were prepared in DI (ρ = 18.2 MΩ) unless otherwise noted.

### Instrumentation

Metrohm Autolab PGSTAT204 electrochemical workstation was utilized for all electrochemical studies using a screen-printed carbon electrode (SPCE) with a three-electrode configuration consisting of a carbon-based working electrode, a counter electrode, and an Ag/AgCl paste reference electrode. The surface morphologies were investigated by using high-resolution transmission electron microscopy (HR-TEM, JEOL JEM-3010). Energy-dispersive spectrometry (EDS) and elemental mapping were performed to establish the elemental composition of synthesized acid-functionalized sp^2^ graphene using a field emission gun transmission electron microscope (FEG-TEM, Tecnai F20 G2MAT S-TWIN). In addition, the successful acid modification of sp^2^ graphene and electropolymerization of aniline were concluded using Fourier transform infrared (FTIR) spectroscopy studies (Thermo Nicolet iS5). Additionally, the uniformity in the morphology of the modified electrodes was inspected by employing field emission scanning electron microscopy (FESEM, JEOL-6330). Finally, the practical feasibility of the developed biosensor chip for real sample analysis was verified using the QuantStudio 5 real-time PCR system based on fluorescence (Thermo Fisher Scientific).

### Sequential Fabrication of HP Sensor

Three-electrode configuration screen-printed carbon electrode (SPCE) with an Ag/AgCl-based reference electrode (RE), a carbon-based working electrode (WE), and a counter electrode (CE) was utilized. Initially, plasma treatment was performed to simultaneously clean and increase the surface hydrophilicity of the WE. Then, acid-modified graphene nanosheets (Gr) with abundant carboxylic groups, synthesized following a previously reported protocol,^30^ were drop-cast and dried at room temperature to achieve SPCE/Gr. Briefly, 20 mg of Gr nanosheets was acid treated using HCl and H_2_SO_4_ at the ratio of 3:1 and thoroughly washed by 5 rounds of centrifugation with DI water at 3000 rpm for 15 min to remove unreacted acids. Then, 2.00 μL of Gr redispersed in DI (1.00 mg/mL) was drop-cast onto plasma-cleansed SPCE to give SPCE/Gr. Subsequently, morphologically distinct Au nanostructures were then electrodeposited on Gr/SPCE from 5.3 mg/mL HAuCl_4_ (aq) in the presence and absence of RE either by (i) linear scanning voltammetry (LSV) at a potential range of −3.00/2.00 to 0.00 V or (ii) chronopotentiometry (CP) for 200 or 250 s at an applied current of −5.00 mA. After the solution was thoroughly washed with DI water, SPCE/Gr/Au was dried at room temperature. Lastly, polyaniline (PANI), in its highly conductive emeraldine form, was electropolymerized under repeated cyclic voltametric scans (potential range: −0.20 to −0.90 V, scan rate: 0.01 V/s) from a solution containing 0.40 mL of aniline and 0.70 mL of H_2_SO_4_ in 25.0 mL of DI. The multilayered electrode was evaluated for morphological homogeneity and electrochemical performance at each stage of the modification process. The fabrication parameters were systematically optimized through detailed characterization and analysis of the resulting morphology and electrochemical behavior to develop the SPCE/Gr/Au/PANI-based electrochemical biosensor chip for monitoring HP infections (discussed in the following sections).

### Morphological and Electrochemical Characterizations

High-resolution transmission electron microscopy (HR-TEM) was employed to analyze the morphology of the as-synthesized acid-modified graphene. In addition, high-angle annular dark-field (HAADF) images, elemental mapping, and Fourier transform infrared spectroscopy (FTIR) were also utilized to analyze the elemental compositions of the graphene nanostructure. Morphological characterizations of the bare and modified electrode surfaces after each modification step were performed by using scanning electron microscopy (SEM). Both top- and cross-sectional micrographs were examined to ensure ideal electrode surface morphology for superior charge-transfer capabilities. Morphologies with high surface areas that demonstrate excellent homogeneity and reasonable reproducibility are desired. Furthermore, enhancements in electrochemical performances, including electroactive surface area and charge-transfer capabilities, were monitored using electrochemical impedance spectroscopy (EIS) and cyclic voltammetry (CV). Equivalent circuit modeling and simulations based on EIS analysis were also performed to decipher the underlying phenomena that dictate the transport of electroactive species at the modified electrode–electrolyte interface (Figure S1 and Table S1). The electroactive surface areas of the electrodes were calculated based on voltametric studies for each of the fabrication steps to gauge and further verify the enhancement in electrochemical performance (Figure S2). Furthermore, the contact angle was recorded to establish the transformations in the hydrophilicity of SPCE/Gr/Au/PANI relative to those of the bare SPCE.

### Sensor Assembly and LAMP Monitoring for HP Detection

The sensing device consisted of the optimized electrode sandwiched between two laser-cut acrylic layers fixed by using locking screws and nuts, as shown in Figure 1. The circular opening of the top acrylic layer, coinciding with the WE surface, is sealed with an O-ring to form an isolated and spill-proof reaction chamber. Additionally, the rectangular cut-out exposes the three electrical connections of the SPCE (corresponding to WE, CE, and RE), which are readily linked to the electrochemical workstation using alligator clips. During LAMP, the increase in amplicons is initiated by primer assimilation via the nucleophilic attack of 3′ −OH on the growing DNA strand. This results in the production of a proton (H^+^) on this terminal and one P_2_O_7_^4–^, which on hydrolysis generates H^+^ and phosphate in equal proportions,^39^ as shown in Figure 1 and eq S1. The released H^+^ leads to pH changes proportional to the amplicon concentrations. The protonation/deprotonation of the PANI layer subjective to [H^+^] was exploited to quantitatively decipher HP DNA concentrations by monitoring the corresponding changes in pH. The detailed protocol that encompasses primer design, formulation of the LAMP reaction mixture, and clinical real-time PCR monitoring is given in the Supporting File (Table S2). Finally, the assembled device was challenged for feasibility for HP detection by monitoring LAMP amplification in two different modes as follows:

(i) Continuous mode (real-time detection): 30.0 μL of the amplification reaction mixture was added to the reaction chamber of the assembled sensor and placed in an oven with a temperature (*T*) > 65.0 °C. Electrical connections with the workstation were established using alligator clips to monitor variations in the generated open-circuit potential (OCP).
(ii) Segmented mode (fixed-point detection): First, the sample was divided into ten tubes, subjected to amplification with an interval of 3.00 min between each tube in a dry bath maintained at a temperature (*T*) > 65.0 °C. The amplified solution was then added dropwise to the sensor reaction chamber. The OCP signal was recorded to establish the degree of amplification and concentration of HP DNA.

**Figure 1:**
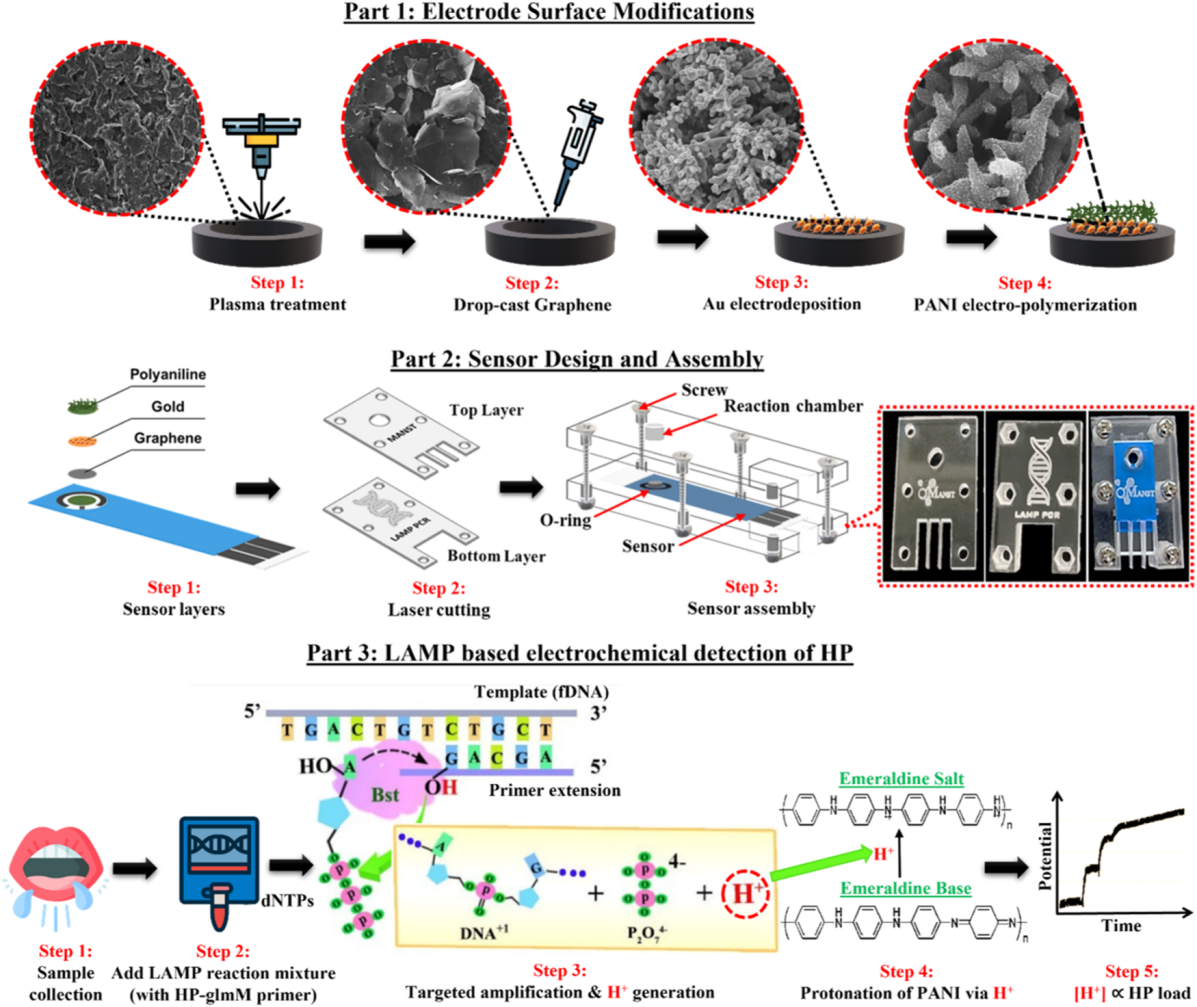
Schematic illustration of the stepwise protocols for (top) electrode modification, (middle) sensor layer assembly, and (bottom) LAMP-based electrochemical detection of HP in biological samples.

### Feasibility for HP Detection in Real Samples

As a proof of concept, the SPCE/Ge/Au/PANI developed method was employed for the detection of HP in artificial saliva. Briefly, filtered and diluted saliva samples were spiked with a known amount of target HP DNA and mixed with the LAMP reaction mixture containing the synthesized LAMP primers targeting the HP glmM gene. Each sample was then subjected to segmented fixed-point detection (as discussed in the section above), and the OCP signal was recorded every 3.00 min using the SPCE/Gr/Au/PANI biosensor chip to establish the degree of amplification and concentration of HP DNA. The final results obtained with the developed biosensor chip were verified using clinical methods (QuantStudio 5, Thermo Fisher Scientific) to determine its feasibility for monitoring the status of HP infections in biological samples with practical precision.

## Results and Discussion

### Characterization of Synthesized Acid-Modified Graphene Nanosheets

The morphological and elemental characterizations of the as-synthesized acid-modified graphene nanosheets were performed using HR-TEM, SEM, and FTIR. The HAADF image in Figure 2(a) and the SEM micrographs in Figure S3­(a) depict the typical 2D sheet morphology of sp^2^ graphene (Gr). The elemental analysis revealed a uniformly distributed presence of carbon and oxygen, suggesting the successful grafting of carboxylic groups (−COOH) onto the surface of the graphene nanosheet, as shown in Figure 2(b–d). This is further supported by the emergence of an FTIR peak at 1720 cm^–1^ in Figure S3­(b), which corresponds to the C=O stretching of-COOH functional groups.^40^ Subsequently, the synthesized graphene with abundant −COOH groups was dispersed in DI, and 2.00 μL (1.00 mg/mL) of this mixture was dropped onto the WE of the SPCE to proceed with additional modification steps.

**Figure 2:**
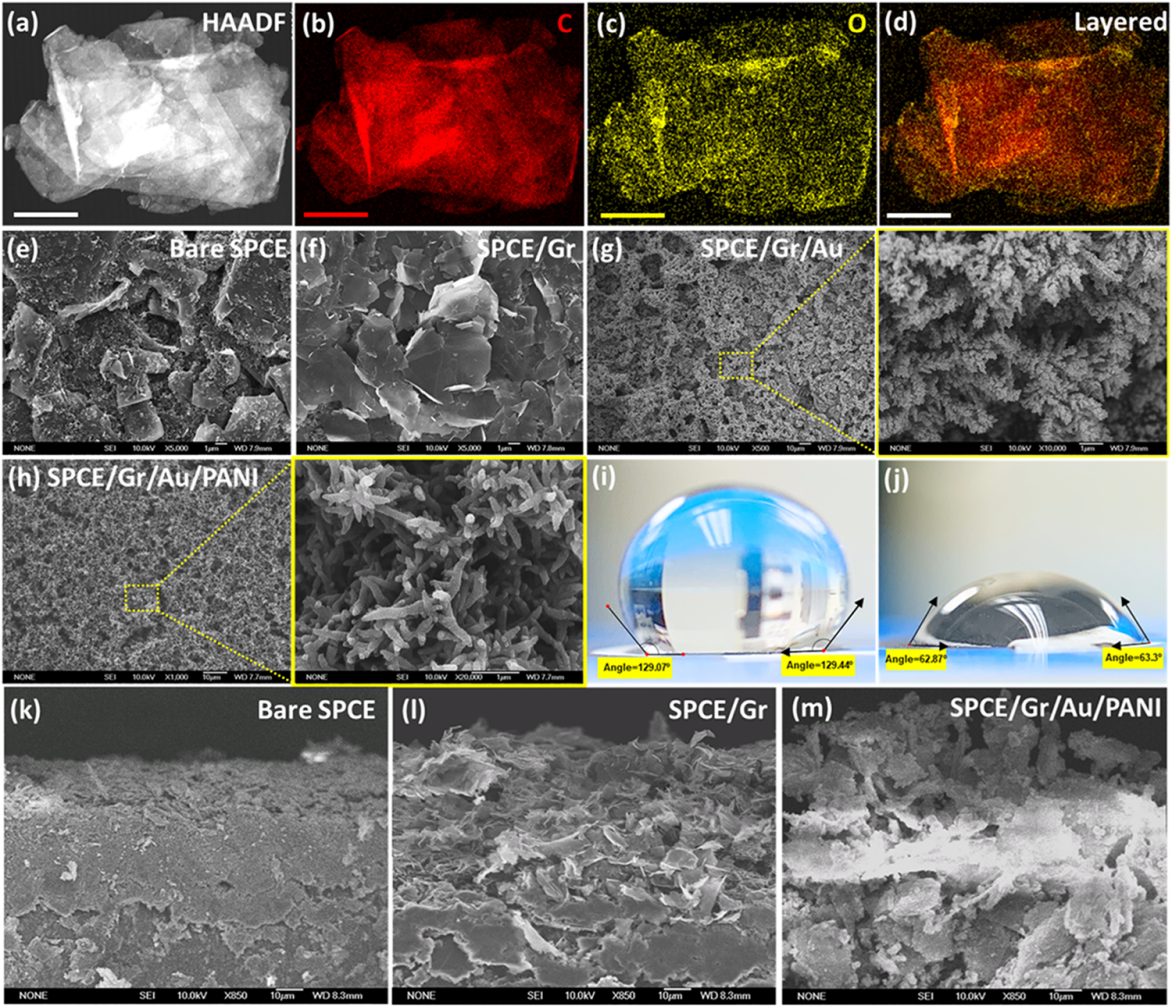
(a) HAADF-TEM image of acid-functionalized graphene. (b–d) EDS elemental maps showing C (red) and O (yellow) distribution [scale bar = 1 μm]. SEM micrographs depicting surface morphologies of (e) bare SPCE, (f) SPCE/Gr, (g) SPCE/Gr/Au, and (h) SPCE/Gr/Au/PANI. (i, j) Contact angle measurements before and after surface functionalization, indicating increased wettability. Cross-sectional SEM images of (k) bare SPCE, (l) SPCE/Gr, and (m) SPCE/Gr/Au/PANI [scale bars as indicated].

### Morphological Optimization of SPCE/Gr/Au/PANI

The splintered surface of the bare electrode in Figure 2(e) was drastically altered following the sequential modification steps, as shown in Figure 2(f–h). Plasma treatment of SPCE improved the hydrophilicity, facilitating efficient anchoring of the sp^2^ Gr nanosheets with the underlying SPCE via the functionalized −COOH groups. This increases the WE surface area through the formation of a lamellar stacked microstructure, as shown in Figure 2(f). Subsequently, premeditated enhancement in charge-transfer capabilities was achieved via electrodeposition of highly conductive Au dendrites, followed by electropolymerization of the pH-sensitive emeraldine form of polyaniline (PANI). Varied parameters for Au electrodeposition via LSV or CP and electropolymerization of aniline via CV were implemented, and the corresponding surface morphologies obtained were examined via FESEM to determine optimal fabrication conditions for superior electrochemical performances.

First, LSV scans were performed from (i) −2.00 V to 0.00 V to (ii) −3.00 to 0.00 V using a two- or three-electrode setup, that is, (A) in the absence and (B) presence of reference electrode (RE). Both method A and method B could achieve deposition results; however, there were obvious differences in the microstructures. At a narrow potential scan range of −2.00 to 0.00 V, nonhomogeneous Au islands with a short grain structure were achieved on the electrode surface (Figure S4). The observed holes or undeposited areas of the electrode were eliminated by widening the potential range from (i) −2.00 to 0.00 V to (ii) −3.00 to 0.00 V, which improved the amount of electrodeposition, as evident from Figure S5. However, a further increase in the potential range (−4.00 to 0.0 V) adversely resulted in an excessively thick Au layer, which easily detaches from the underlying electrode surface. Alternatively, employing the RE during LSV-based electrodeposition of Au (using the same solution and parameters) drastically improved the overall surface morphology, regardless of the scan range. The participation of the reference electrode, as expected, enabled relatively stable variations in electrical potential during LSV.^41^ Consequently, the uniformity and degree of deposition of the Au layer improved significantly. In addition, the microstructure showed a complete transformation from grain-like to needle-like dendritic 3D microstructures, which is ideal to increase the electroactive surface area, as shown in Figures S6–S7 and 2(g).

Second, Au was also deposited using CP (with or without RE) with a fixed applied current of −5.00 mA and a deposition time of 200 and 250 s. In the absence of RE, blunted dendritic structures were observed, which, with increasing deposition time, progressively aggregated, as shown in Figures S8–S9. Such a morphology is not expected to show ideal electrochemical properties. Moreover, the participation of RE did not have a significant difference in the nanostructure and amount of deposition, as shown in Figures S10–S11. This is attributed to the independence of the deposition kinetics on applied potential during CP, wherein the deposition is performed at a fixed potential state while varying only the deposition time, thereby eliminating the influence of the RE. Additionally, the relative increase in Au aggregation on the electrode surface led to a nonhomogeneous distribution, which is not ideal for improving electrochemical capabilities.^42^ Therefore, based on initial morphological analysis, Au electrodeposition using LSV at −3.00 to −0.00 V in the presence of RE was selected as the optimal fabrication conditions. Additional electrochemical characterizations were performed to substantiate this interpretation (discussed in the following section).

Finally, aniline was electrochemically polymerized on the SPCE/Au instrument via repeated CV scans of up to seven cycles. Polymerization cycles were limited to seven cycles since further increments led to a thicker PANI layer, which flakes out of the electrode surface. FTIR analysis of SPCE/Gr/Au/PANI shown in Figure S3­(b) revealed N–H stretching peak at 3010 cm^–1^, indicating successful PANI polymerization on the electrode surface.^43^ The presence of RE facilitated a stable variation in potential with a precisely sustained scan rate that significantly impacted the final morphology, as shown in Figures S12–S13. The aggregated granular microstructure observed in Figure S12 has been effectively transformed into a well-defined pointed fibrillar morphology (Figure S13). The fibrillar PANI shown in Figure 2(h) was used as the pH-sensitive layer of the biosensor chip by exploiting its efficient doping/dedoping characteristics to enable fast, cost-effective, and reversible (bidirectional) pH monitoring.^44^ Furthermore, Figure 2(i–j) shows a decrease in the average contact angle from 129.25 to 63.08°, suggesting considerable improvement in hydrophilicity. In addition, cross-sectional SEM micrographs of the bare SPCE and optimally modified electrodes in Figure 2(k–m) reveal significantly different morphologies. SPCE/Gr/Au/PANI demonstrated homogeneity and high surface area owing to fibrillar PANI-encapsulated Gr nanosheets, which are ideal for enhancing electrochemical properties. The relationship between the fabrication parameters, the optimally selected morphologies, and the corresponding electrochemical performances (accessed in terms of sensitivity and response time) is explored in the next section.

### Electrochemical Optimization of SPCE/Gr/Au/PANI

The FESEM analysis suggested ideal electrode surface morphologies under certain fabrication parameters. This necessitated further electrochemical analysis to determine optimal fabrication conditions for the development of the proposed highly sensitive biosensor chip. To this end, the overall charge-transfer properties of the modified electrodes in terms of EASA, peak redox, current, and sensitivity to detect pH variations were examined.

First, CV was performed using each of the electrodes developed under various fabrication conditions. As expected, these electrodes with distinct Au and PANI microstructures demonstrated varied charge-transfer behaviors, as shown in Figure 3(a). pH-sensitive PANI complemented by the conductivity of the underlying dendritic Au-covered graphene nanosheets translated into amplified charge-transfer capabilities, thus demonstrating the highest peak redox current.^45^ Second, this hypothesis was further substantiated through EIS analysis, which manifested as the progressively decreasing semicircles in the Nyquist plots in Figure 3(b), indicating decreased resistance to charge transfer (*R*_ct_).^46^ Also, PANI polymerization can be followed from the voltammograms in Figure 3(c). And third, the percentage of normalized current variation, Δ*I*% (calculated as (*I* – *I*_0_)/*I*_0_ × 100, where *I* and *I*_0_ are the oxidation peak current of SPCE and the modified electrode, respectively), for each electrode was compared. Figure 3(d–e) revealed enhanced electrochemical performance for electrodes subjected to Au layer electrodeposition via LSV (−3.00 V to −0.00 V in the presence of RE), as evident from the high Δ*I*% with negligible error. Thus, LSV was chosen over CP for Au electrodeposition. Furthermore, PANI via CV (0.20 V to −0.90 at 0.01 V/s) showed an improvement in sensitivity to pH variations with increasing polymerization cycles (*n*), as shown in Figure 3(f), presumably due to the higher degree of aniline polymerization at the electrode surface. The highest pH performance was observed at *n* = 7, as a further increase resulted in unwanted, excessively thick PANI. Figure 3(g,j) shows the feasibility of modified electrodes for repeated pH monitoring. Highly consistent results with ideal sensitivity and reproducibility were observed for SPCE/Gr/Au/PANI developed via LSV (−3.00 to 0.00 with RE) and PANI with *n* = 7, and therefore it was concluded that it was the ideal fabrication parameters.

**Figure 3:**
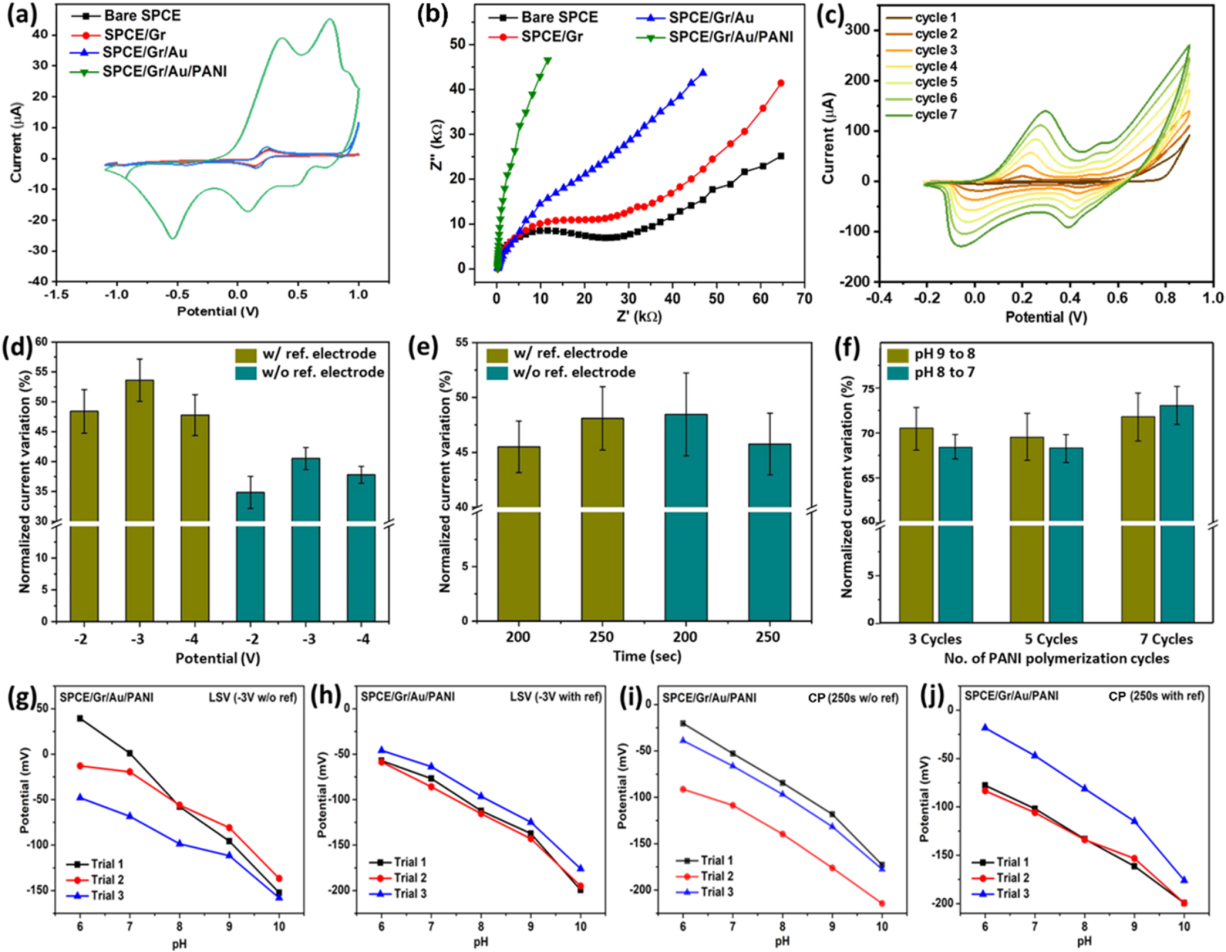
(a) Cyclic voltammograms recorded in 5.0 mM [Fe­(CN)_6_]^3–^/^4–^ redox probe (scan rate = 0.01 V/s), highlighting the progressive enhancement in redox peak currents with each electrode modification. (b) Nyquist plots from EIS measurements indicating improved electron transfer kinetics upon sensor fabrication. (c) Electrochemical polymerization of aniline via cyclic voltammetry. Optimization of Au electrodeposition conditions using (d) LSV within potential windows of −2.00 to 0.00 V, −3.00 to 0.00 V, and −4.00 to 0.00 V, and (e) CP at −5.00 mA for 200 and 250 s. (f) Response reproducibility assessed by normalized current variation under cyclic pH shifts (pH 9 ↔ 8) with increasing electropolymerization cycles. Repetitive pH sensing (*n* = 3) using SPCE/Gr/Au/PANI electrodes fabricated via (g) LSV at −3.00 V (without reference), (h) LSV at −3.00 V (with reference), (i) CP at 250 s (without reference), and (j) CP at 250 s (with reference).

Furthermore, equivalent circuit modeling and simulations based on the Randles circuit in Figure S1 confirmed the close agreement between the observed and simulated Nyquist plots. This concluded the faithful representation of the transport phenomena of electroactive species at the electrode–electrolyte interface.^47^ Furthermore, the observed R_ct_ value extracted from the simulations was found to be the lowest for SPCE/Gr/Au/PANI owing to the introduction of synergistic microstructures, indicating excellent relative charge-transfer efficiency (Table S3).

### Bidirectional pH Sensing Capabilities

Figure 4(a) shows the electrochemical responses of the modified electrodes to the variation in pH (9.0 to 8.0 in this case). Inherent differences in the composition of the sensing layer in each of the distinctly modified electrodes resulted in varied starting potentials. Thus, to eliminate possible inaccuracies, a simple yet effective form of baseline correction was used. The percentage normalized potential variation (Δ*P*%) was calculated as |(*P* – *P*_0_)/*P*_0_ × 100|, wherein *P*_0_ and *P* are potentials at the initial and final pH, respectively. Figure 4(b) presents the calculated Δ*P*% of SPCE/PANI, SPCE/Gr/PANI, SPCE/Au/PANI, and SPCE/Gr/Au/PANI and their respective response time. The quickest response time and highest sensitivity to pH variation were observed using the optimized SPCE/Gr/Au/PANI. It also demonstrated excellent feasibility for bidirectional monitoring, i.e., both positive and negative pH variations, with outstanding repeatability and reproducibility, as shown in Figure 4(c). The decrease in pH, that is, the increase in [H^+^], resulted in the protonation of PANI, which transformed it from its emeraldine base form to its corresponding emeraldine salt form.^48^ This led to increased conductivity, thus resulting in a resolvable increase in potential and vice versa.^49^ Therefore, an inverse relationship was established between pH and the observed potential with high linearity (*R* ^2^ = 0.989), as shown in Figure 4(d). Synergistic collaborations between pH-responsive PANI, highly conductive Au dendrites, and sp^2^ graphene translated to a highly electroactive surface microstructure with superior pH sensitivity and charge-transfer capabilities.^50^ Thus, the practical feasibility of the developed biochip for continuous and reliable pH monitoring applications was established. Consequently, the SPCE/Gr/Au/PANI-based biochip was then utilized for the quantitative verification of HP infection status by monitoring the pH changes associated with LAMP amplification of the target HP DNA via two modes, namely, the continuous mode and the segmented mode.

**Figure 4:**
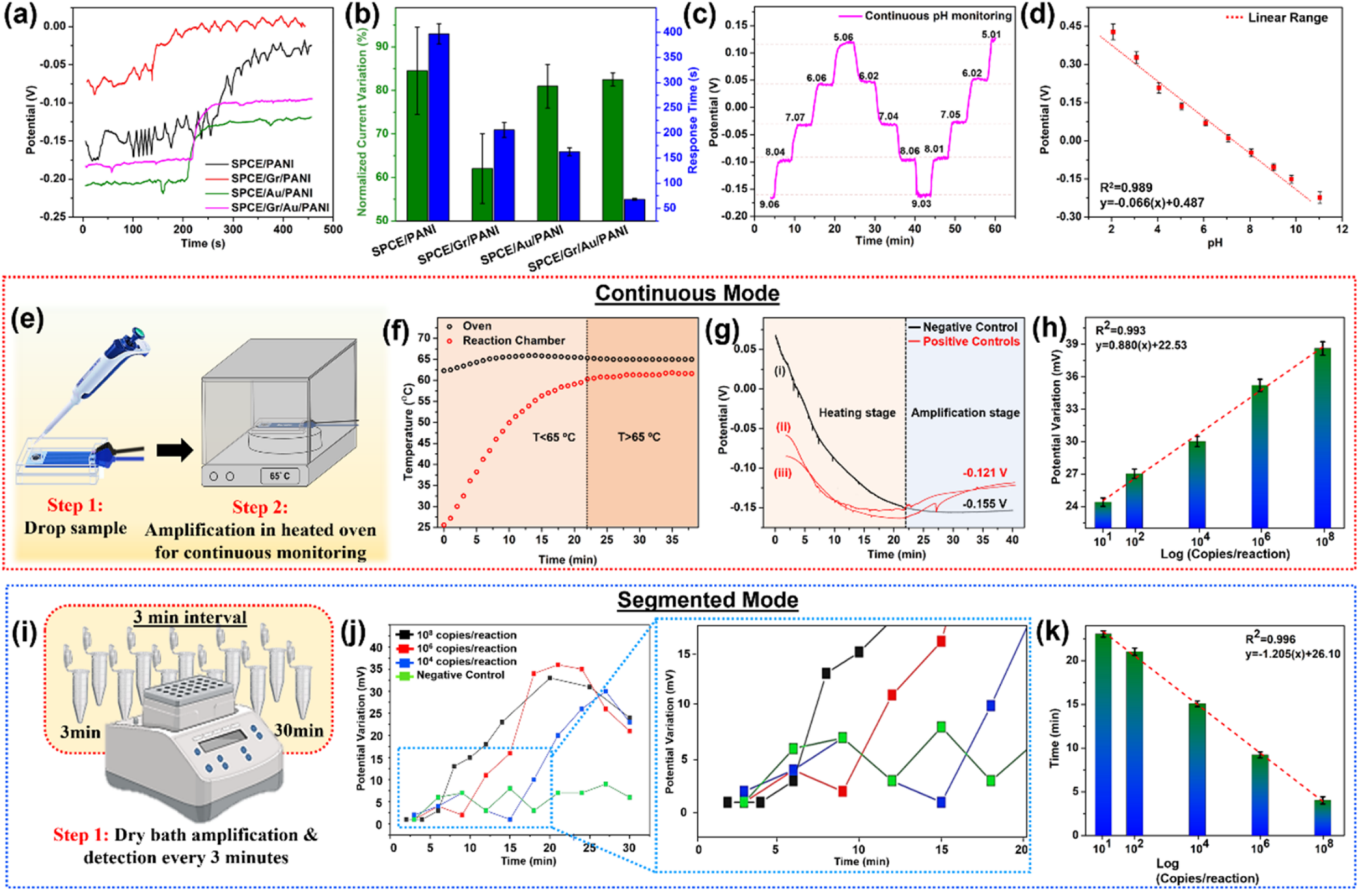
(a) Potential–time response demonstrating electrode sensitivity to pH changes. (b) Comparison of potential variations and response time. (c) Bidirectional continuous pH monitoring (pH 5–9). (d) Calibration curve showing linearity between pH and potential (*R* ^2^ = 0.989). (e) Schematic for continuous-mode HP detection. (f) Thermal profile showing chamber equilibration at 65 °C (*t* = 22 min). (g) Continuous potential shifts for varying HP DNA concentrations: (i) negative control, (ii) 1, (iii) 10^4^, and (iv) 10^7^ copies/μL. (h) Calibration curve correlating potential with HP DNA concentration (*R* ^2^ = 0.993). (i) Schematic of segmented detection mode. (j) Segmented potential variation with HP DNA concentrations. (k) Calibration curve showing time (*t*) to achieve Δ*P* ≥ 10 mV versus initial DNA concentration (*R* ^2^ = 0.996) [all error bars: SD, *n* = 5].

### RT-LAM Augmented Electrochemical Detection of HP Using SPCE/Gr/Au/PANI

In continuous monitoring mode, as shown in Figure 4(e), the temperature (*T*) of the oven and reaction chamber must be equilibrated to *T* = 65 °C to initiate the amplification process. The time-dependent thermal plot in Figure 4(f) indicates that the threshold temperature of 65 °C was achieved after a time (*t*) of 22.00 min. Consequently, the amplification of HP DNA via LAMP was continuously monitored using the assembled biosensor chip (Figure 1) and reported a gradual increase in potential after 22.00 min, which eventually plateaus. Therefore, the higher initial concentration of HP target DNA led to a higher potential plateau, as shown in Figure 4(g). A calibration graph was established that correlates the HP target DNA concentration, and the final potential was established with good linearity (*R* ^2^ = 0.993) in Figure 4(h). Therefore, HP can be detected and quantified through continuous monitoring of [H^+^] generated during the amplification stage. However, the slope of the calibration curve suggested a reduced sensitivity (*s* = 0.850 mV), presumably due to heating inefficiency in the reaction chamber and the possible interaction of the reaction mixture with the electrode surface.^51^

Alternatively, an enhancement in sensitivity was explored using the fixed/segmented detection mode, as shown in Figure 4(i). Predictably, the potential increases over time because of [H^+^] generated during amplification. An abrupt exponential increase in potential (Δ*P*) was observed at varying time periods (*t*) for different starting HP DNA concentrations, as shown in Figure 4(j). The amplification time required to surpass the threshold of Δ*P* ≥ 10.00 mV was found to be inversely proportional to the initial HP DNA concentration. Therefore, the value of “*t*” at which Δ*P* ≥ 10.00 mV was individually translated to the initial HP DNA concentration of HP (copies/μL). A highly linear detection range with improved sensitivity (*s* = 1.205 mV) was achieved, as shown in Figure 4(j).

Additionally, to evaluate the specificity of the key primers developed in this study (F2, B3, F1c, and B1c), we performed sequence alignment against a genetic database. The search included bacterial species known to coexist with HP, such as *Campylobacter jejuni*, *Vibrio parahemolyticus*, and *Vibrio cholerae*. A summary of these statistical results is provided in Table S4, which exclusively lists species for which the Basic Local Alignment Search Tool (BLAST) analysis returned a significant alignment. The alignment results demonstrate a high degree of specificity; the *E*-values for all potential cross-reactions with nontarget species were greater than 0.05, indicating no cross-reactivity.

### Real Sample Test and Clinical Verification

As a proof of concept, the fabricated biosensor chip was utilized to quantify HP in biological samples, and the results were verified by fluorescence-based clinical quantification using the QuantStudio 5 real-time PCR system (sample preparation is detailed in the Supporting Information file). The amplification status was recorded every 3 min by monitoring the change in potential (Δ*P*) with time (*t*), as shown in Figure 5(a). The HP initial DNA concentration (copies/μL) for each of the three positive saliva samples and one negative control was deduced using the calibration graph in Figure 4(k) and simultaneously using the fluorescence-based real-time PCR system. It is worth mentioning that the potential change and fluorescence quantification in Figure 5(b–e) exhibit a mutually dependent trend with only a slight deviation at some points. However, it should be noted that the potential measurements and fluorescence quantification at every segment of each concentration demonstrated the same trend, including the aforementioned deviations. Furthermore, the excellent correlation between the electrochemical and fluorescence results for each positive saliva sample shown in Figure 5(f) and the excellent recovery (%) in Table S5 conclude the practical feasibility of the developed sensor to monitor HP infections in biological samples with reliable clinical precision. Finally, Table 1 presents a comparison table highlighting the achievements and improvements of the current biochip over existing HP detection technologies.

**Figure 5:**
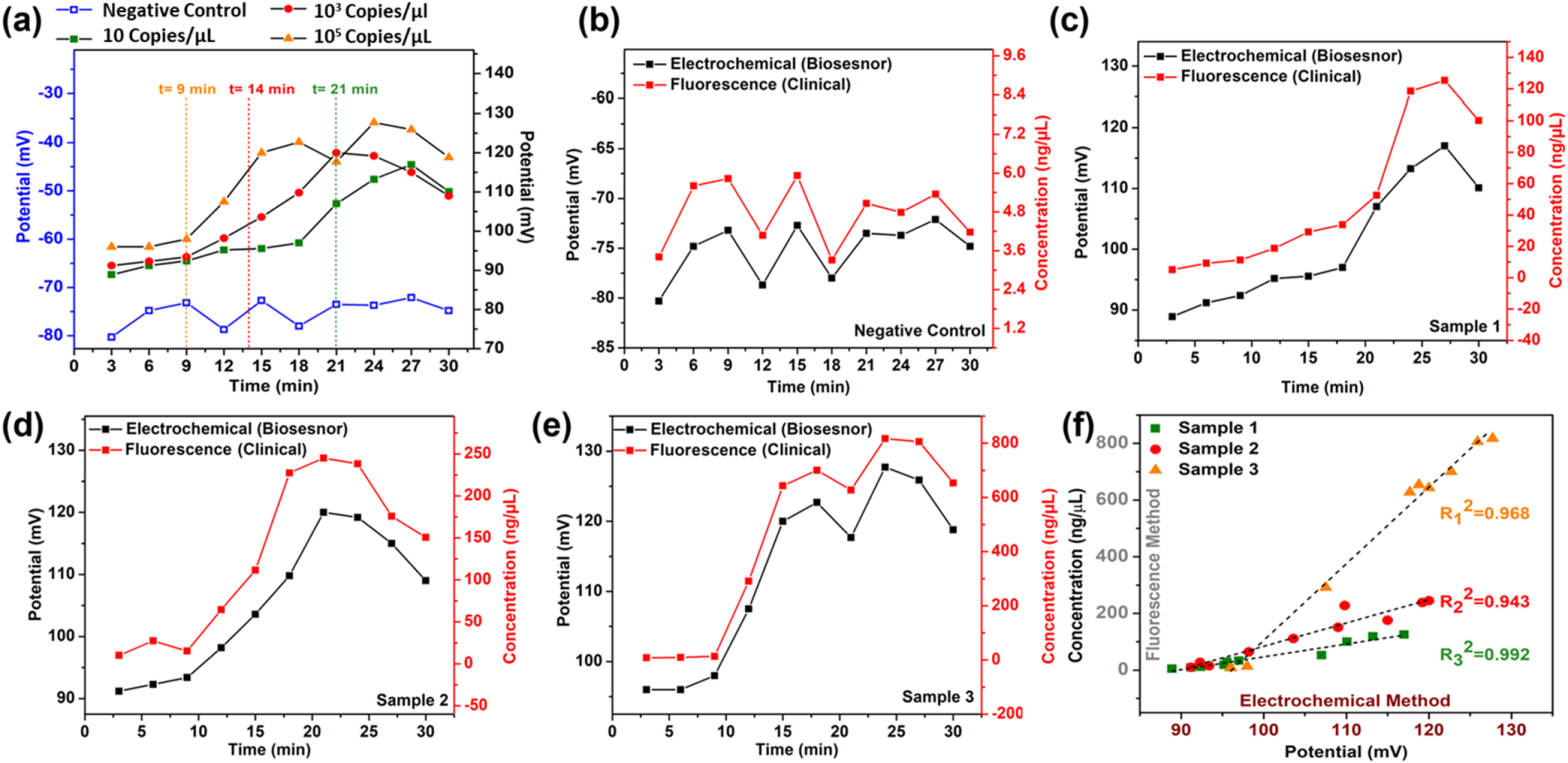
(a) Detection of HP DNA in spiked biological samples. A comparison of biosensor output with fluorescence data for (b) negative control; (c–e) samples 1–3. (f) Linear regression analysis showing a strong correlation between biosensor and clinical results.

**Table 1:** Comparison Table Highlighting the Advantages of the Proposed Biochip over Existing HP Detection Technologies

method	biomarker	mechanism	sample	detection range	LOD	assay time	operational complexity	robustness	limitations	refs
EC	HopQ protein	Ab-Ag binding	drinking water	5 to 1063 pg/mL	2.06 pg/mL	>30 min	moderate (post-incubation washing required)	low (not validated in biological samples)	unverified protein interference	^35^
CL	NH_3_	pH (color change)	artificial gastric fluid	1 to10 CFU/mL	1 CFU/mL	3 h	moderate (filtration needed)	moderate (Invasive sampling)	smartphone camera sensitivity affects accuracy	^36^
FL/CRISPR/Cas	Genomic DNA	HSEA	purified DNA extract	20 to 220 ng/ μL	70 ng/μL	<1.5 h	high (gastric mucosa sampling)	moderate	cross-fluorescence observed	^37^
Fl/LAMP	Virulence gene	Turn-on (calcein)	biopsy samples	10^5^ to 10 ng/μL	1 pg/μL	1 h	high (biopsy sampling)	high (tested in patients)	semiquantitative visual verification	^38^
EC/LAMP	glmM gene	[H^+^] tracking	saliva	1 to 10^7^copies/μL	1 copy/μL	>30 min	low (facile assay and handling)	high (validated in noninvasive sample)	minor risk from post-amplification handling	this work

## Conclusions

In summary, a portable and inexpensive biosensing platform with a two-mode approach was successfully developed for the quantitative detection and monitoring of HP infection through pH tracking of LAMP-based HP DNA amplicon growth. The multilayer biochip constructed with a three-electrode system configuration consisted of an underlying SPCE sequentially modified with acid-functionalized sp^2^ graphene, electrodeposited needle-like Au dendrites, and finally encapsulated with electropolymerized PANI in its pH-sensitive emeraldine form. This synergistic architecture provided a large electroactive surface area, thereby enhancing the biosensors’s overall electrochemical performance. Consequently, sensitivity of (i) 0.880 mV (LOD = 1 copy/μL) and (ii) 1.205 mV (LOD = 1 copy/μL) were obtained by continuous and segmented detection modes, respectively, both in the sensing range of 1 to 10^7^ copies per reaction. Furthermore, the lowered sensitivity associated with prolonged interactions of the reaction mixture with the PANI microstructure was effectively mitigated by employing the segmented detection mode. This allowed enhanced sensitivity by reducing the interaction time between PANI and the reaction mixture. Finally, excellent correlation with clinical gold standard instruments and high recovery (%) in spiked biological samples confirmed the practical feasibility of the proposed dual-mode LAMP-augmented electrochemical biosensing platform for POC monitoring of HP infection status.

## Supplementary Material



## Notes

Electroactive surface area calculation; HP primer design and synthesis; mechanism of LAMP-induced pH variation; saliva sample preparation protocol; equivalent circuit modeling and simulation; cross-reactivity analysis via database alignment; electroactive surface area of bare and modified electrodes; SEM of –COOH functionalized graphene; FTIR spectra of aniline, SPCE/Gr (acid-modified), and PANI on SPCE/Gr/Au; SEM optimization of Au electrodeposition using LSV (−2.00 to 0.00 V, −3.00 to 0.00 V) and chronopotentiometry (−5.00 mA, 200 to 250 s) under both the presence and absence of reference (Ag/AgCl) configurations (PDF)

## Abbreviations

EC: electrochemical
FL: fluorescence
CL: colorimetric
CRISPR/Cas: clustered regularly interspaced short palindromic repeats (CRISPR)/CRISPR-associated (Cas) system
LAMP: loop-mediated isothermal amplification
Ab-Ag: antibody–antigen
HSEA: hairpin-mediated self-primer exponential amplification
CFU: colony-forming unit
