# Supplementary material for: Hierarchical Graphene/Au/Polyaniline Nanostructured Electrode for Dual-Modality Electrochemical LAMP Biosensing of Helicobacter pylori
Source: Anal Chem. 2025 Nov 4;97(45):25148–57. doi: 10.1021/acs.analchem.5c04200 (PMC12631725; doi:10.1021/acs.analchem.5c04200)
Supplement: Supplementary file 1 [file ac5c04200_si_001.pdf]

Supporting Information

Hierarchical Graphene/Au/polyaniline nanostructured electrode for dual-modality electrochemical LAMP biosensing of Helicobacter pylori

Rajkumar Rakesh Kumar [a,b†], Chi-Yu Wang [c†], Aditya Manu Bharti [a,f], Wen-Chian Hu [a], Yi-Chen Zhang [a], Ling-Shan Yu [d], Pornnapat Tungtrakarnkul [e], Kevin C.-W. Wu [b*] , Cheng-Hsin Chuang [a*]

[a] Institute of Medical Science and Technology, National Sun Yat-sen University, Kaohsiung 80424, Taiwan.

[b] Institute of Biomedical Engineering and Nanomedicine, National Heath Research Institutes, Miaoli 350401, Taiwan.

[c] Medical Cosmetic Institution, Kaohsiung Armed Forces General Hospital, Kaohsiung 802301, Taiwan.

[d] Institute of Biopharmaceutical Science, National Sun Yat-sen University, Kaohsiung 80424, Taiwan.

[e] Department of Biomedical Engineering, Mahidol University, Nakhon Pathom 73170, Thailand.

[f] International PhD Program for Science, National Sun Yat-sen University, Kaohsiung 80424, Taiwan.

[†]Co-first Authors

Corresponding author Email: chchuang@imst.nsysu.edu.tw

Table of Contents

Calculation of the electroactive surface area **S3**

Table S1: Calculated EASA at each stage of the modification process **S3**

Helicobacter pylori Primer Design and Synthesis **S3**

Table S2: Helicobacter pylori LAMP primer sequences **S4**

pH variations during Loop-mediated isothermal amplification (LAMP) **S5**

Saliva sample preparation for HP detection **S5**

Equivalent Circuit Modeling and Simulation **S6**

Figure S1: Circuit Design and simulation **S6**

Table S3: Simulation results based on the equivalent circuit model analysis. **S7**

Assessment of Cross-Reactivity Using Database Alignment **S7**

Table S4: Basic Local Alignment Search Tool (BLAST) analysis. **S7**

Figure S2: Electroactive Surface Area **S10**

Figure S3: Functionalization **S11**

Figure S4: Au electrodeposition (LSV: -2.00 V to 0.00 V, w/o Ag/AgCl) **S11**

Figure S5: Au electrodeposition (LSV: -3.00 V to 0.00 V, w/o Ag/AgCl) **S12**

Figure S6: Au electrodeposition (LSV: -2.00 V to 0.00 V, w/ Ag/AgCl) **S12**

Figure S7: Au electrodeposition (LSV: -3.00 V to 0.00 V, w/ Ag/AgCl) **S13**

Figure S8: Au electrodeposition (CA: -5.00 Ma, 200 s, w/o Ag/AgCl) **S13**

Figure S9: Au electrodeposition (CA: -5.00 Ma, 250 s, w/o Ag/AgCl) **S14**

Figure S10: Au electrodeposition (CA: -5.00 Ma, 200 s, w/ Ag/AgCl) **S14**

Figure S11: Au electrodeposition (CA: -5.00 Ma, 250 s, w/ Ag/AgCl) **S15**

Figure S12: PANI electro polymerization w/o Ag/AgCl **S15**

Figure S13: PANI electro polymerization w/ Ag/AgCl **S16**

Table S5: HP quantification in spiked biological samples **S16**

Calculation of the electroactive surface area

The electroactive surface area (EASA) of the bare SPCE and the modified electrodes at each stage of the fabrication step was determined by performing CV in 5 mM $[Fe(CN)_6]^{2-}/[Fe(CN)_6]^3$ within the potential range of -1.20 V to 1.00 V at a scan rate of 0.01 V/s. The corresponding peak oxidative currents were extracted from Figure 3(a) to calculate the value of EASA using the Debye-Scherrer equation as given below,

$$\mathbf{Ip} = 268600\boldsymbol{n}^{(3/2)}\mathbf{A}\mathbf{D}^{(1/2)}\mathbf{C}\boldsymbol{v}^{(1/2)}$$

Ip (in A) is the peak current, ***n*** is the number of electrons transferred in the redox reaction, **A** (in cm^2) is the electroactive surface area, **D** (in cm^2/s) is the diffusion coefficient, **C** (in mol/cm^3) is the concentration of the reactive species in the electrolyte, and ***v*** (in V/s) is the scan rate.

Table S1: Calculated EASA at each stage of the modification process.

**Electrode**	**Peak current (A)**	**Electroactive Surface Area (cm^2)**
SPCE	2.30E-06	6.99E-03
SPCE/Gr	3.10E-06	9.42E-03
SPCE/Gr/Au	4.50E-06	1.37E-02
SPCE/Gr/Au/PANI	3.97E-05	1.21E-01

Helicobacter pylori Primer Design and Synthesis

LAMP primers targeting the Helicobacter pylori (HP) glmM gene were designed using PrimerExplorer V5 software (Eiken Chemical Co. Ltd., Tokyo, Japan; https://primerexplorer.jp) and manually adjusted based on simulation results obtained from NUPACK

(https://www.nupack.org/). The primer sets consisted of two outer primers (F3 and B3), two inner primers (FIP and BIP), and two loop primers (LF and LB), as detailed in **Table S2**.

The synthetic target DNA of Helicobacter pylori was synthesized by Integrated DNA Technologies (The Netherlands). The LAMP reaction mixtures were prepared as follows: 1 × optimized isothermal buffer (pH 8.8), 6 mM $MgSO_4$ (New England Biolabs, USA), 1.4 mM dNTP (Promega, USA), 1.2 mg / ml bovine serum albumin (New England Biolabs), 0.8 M betaine (Sigma, USA), 1 × LAMP primer mix (Integrated DNA Technologies), 0.5 μM Syto 9 (Thermo Fisher Scientific), 0.32 U/μL Bst 2.0 DNA polymerase (New England Biolabs), a synthesized DNA solution ranging from 10^7 to 1 genomic copies/μL, and nuclease-free water to adjust the total reaction volume to 10 μL. The LAMP reaction was monitored in real time using a QuantStudioTM 5 real-time PCR system (Thermo Fisher Scientific).

Table S2: Helicobacter pylori LAMP primer sequences

**Primer name**	**Sequence (5′–>3′)**
**F3**	AACCCCTAACACCCCTAA
**B3**	GTTTTAGTGATTAATGATGAGCCTA
**FIP**	CTTTGCTTTTGATGGCGATGCCCATGCACGATATTCCCT
**BIP**	CCAAATCCGCGCGGTATTTTGTGTAATATTAATGAGCAATGCG
**LF**	AGGCTAGTGGTGGTGGATAATTT
**LB**	TTCACTTCCTGGCTCAACTGG

pH variations during Loop-mediated isothermal amplification (LAMP)

Loop-mediated isothermal amplification (LAMP) inherently releases hydrogen ions (H^+). The reaction equation can be represented as

$$\text{dNTPs} + \text{DNA polymerase} \rightarrow \text{DNA} + \text{PPi} + 2H^+ \qquad \textbf{(equation S1)}$$

where, dNTPs: Deoxynucleotide triphosphates, which are the building blocks of DNA.

DNA polymerase: An enzyme that catalyzes DNA synthesis.

DNA: The newly synthesized DNA strand.

PPi: Pyrophosphate, a byproduct of the reaction.

$2H^+$: Two hydrogen ions were released for each deoxynucleotide incorporated into the DNA.

Saliva sample preparation for HP detection

Artificial saliva samples were used directly without further processing. 20µL aliquots of saliva sample aliquots were mixed with 1 × optimized isothermal buffer (pH 8.8), 6 mM MgSO4 (New England Biolabs, USA), 1.4 mM dNTP (Promega, USA), 1.2 mg/mL bovine serum albumin (New England Biolabs), 0.8 M betaine (Sigma, USA), 1 × LAMP primer mix (Integrated DNA Technologies), 0.5 µM Syto 9 (Thermo Fisher Scientific), 0.32 U/µL Bst 2.0 DNA polymerase (New England Biolabs), a synthesized DNA solution of desired concentration (genomic copies/reaction volume), and nuclease-free water to adjust the total volume to 30 µL. This reaction mixture consisting of spiked HP DNA was then subjected to the proposed detection via segmented and continuous mode. The observed concentration and the known concentration were compared to validate the accuracy of the developed HP detection biosensing platform.

Equivalent Circuit Modeling and Simulation

Nyquist plots corresponding to the EIS analysis of the bare SPCE and the modified electrodes at each stage of the fabrication, i.e., SPCE/Gr, SPCE/Gr/Au, and SPCE/Gr/Au/PANI, were constructed. The observed trend was fitted according to a modified Randles circuit, consisting of four electrical components namely (i) resistance of the solution (R_s), (ii) double layer capacitance (C_{dl}), (iii) resistance to charge transfer (R_{ct}), and (iv) Warburg impedance (W) as shown in **figure S1(a)**. The observed Nyquist plot is in close agreement with the simulated data as shown in **figure S1(b-e)**, thus establishing legitimacy of the proposed model to accurately portray the electrochemical properties of the modified electrode. Furthermore, R_s, C_{dl}, R_{ct}, and W calculated based on the equivalent circuit model analysis are listed in **Table S3**.

Figure S1. Circuit Design and simulation: (a) Equivalent circuit modeled to represent the transport phenomena of electroactive species at the electrode-electrolyte interface. Comparative Nyquist plot showing the observed result of the EIS analysis plotted with their respective simulated data corresponding to (b) bare SPCE, (c) SPCE/Gr, (d) SPCE/Gr/Au, and (e) SPCE/Gr/Au/PANI.

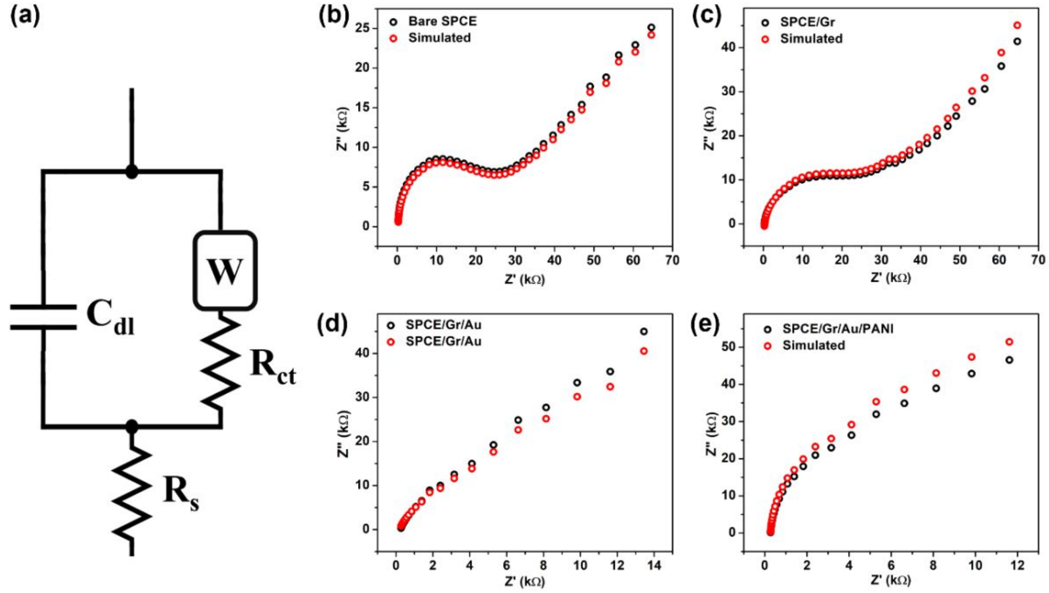

Table S3: Table listing the values of resistance of the solution (R_s), double layer capacitance (C_{dl}), resistance to charge transfer (R_{ct}) and Warburg impedance (W) of the bare and modified electrodes based on the equivalent circuit model analysis.

Electrode	R_s (Ω)	R_{ct} (kΩ)	C_{dl} (μ℧s^N)	W (μ℧s$^{1/2}$)
Bare SPCE	125.00	30.23	2.09 (N=0.79)	425.00
SPCE/Gr	128.00	25.08	8.71 (N=0.81)	358.00
SPCE/Gr/Au	125.00	11.26	13.85(N=0.93)	240.00
SPCE/Gr/Au/PANI	126.00	4.07	24.6 (N=0.68)	201.00

Assessment of Cross-Reactivity Using Database Alignment

To evaluate the specificity of the key primers developed in this study (F2, B3, F1c, and B1c), we performed a sequence alignment against a genetic database. The search included bacterial species known to coexist with *Helicobacter pylori*. The alignment results demonstrate a high degree of specificity; the E-values for all potential cross-reactions with non-target species were greater than 0.05. A partial summary of these statistical results is provided in the table below.

Table S4: The table exclusively lists species for which the Basic Local Alignment Search Tool (BLAST) analysis returned a significant alignment. Species with no significant similarity are not reported by the algorithm

Viral strain	Accession number	E-value				Detectability
		Primer F2	Primer F1c	Primer B2	Prime r B1c	
Campylobacter jejuni	CP010306.1	845	0.25	42	n.a	Will not be detected

Campylobacter jejuni	CP125739.1	845	0.25	42	n.a	Will not be detected
Campylobacter jejuni	CP022079.1	845	0.25	42	n.a	Will not be detected
Campylobacter jejuni	CP125741.1	845	0.25	42	n.a	Will not be detected
Campylobacter jejuni	CP148238.1	845	0.25	42	n.a	Will not be detected
Campylobacter jejuni	CP048760.1	253	0.25	253	n.a	Will not be detected
Campylobacter jejuni	CP048769.1	253	0.83	42	845	Will not be detected
Campylobacter jejuni	CP126353.1	253	0.25	42	845	Will not be detected
Campylobacter jejuni	CP054848.1	n.a	0.25	253	845	Will not be detected
Campylobacter jejuni	CP048765.1	n.a	0.83	42	845	Will not be detected
Citrobacter freundii	CP114565.1	863	541	n.a	n.a	Will not be detected
Citrobacter freundii	CP110779.1	863	n.a	n.a	n.a	Will not be detected
Citrobacter freundii	CP057509.1	216	136	48	27	Will not be detected
Citrobacter freundii	CP133060.1	54	34	n.a	n.a	Will not be detected
Citrobacter freundii	CP137117.1	54	34	758	433	Will not be detected
Citrobacter freundii	LR881934.1	54	34	758	109	Will not be detected
Citrobacter freundii	CP133060.1	54	34	n.a	n.a	Will not be detected

Citrobacter freundii	CP137128.1	54	34	190	109	Will not be detected
Citrobacter freundii	CP135452.1	n.a	541	n.a	n.a	Will not be detected
Citrobacter freundii	CP040696.1	n.a	n.a	758	n.a	Will not be detected
Vibrio parahaemolyticus	CP078693.1	308	3.0	232	616	Will not be detected
Vibrio parahaemolyticus	CP078647.1	308	3.0	232	616	Will not be detected
Vibrio parahaemolyticus	CP078631.1	308	3.0	232	616	Will not be detected
Vibrio parahaemolyticus	CP047990.1	308	3.0	n.a	154	Will not be detected
Vibrio parahaemolyticus	CP187430.1	308	48	232	n.a	Will not be detected
Vibrio parahaemolyticus	CP129936.1	77	3.0	924	616	Will not be detected
Vibrio parahaemolyticus	CP114186.1	77	3.0	n.a	154	Will not be detected
Vibrio parahaemolyticus	CP078646.1	n.a	193	232	n.a	Will not be detected
Vibrio parahaemolyticus	CP187438.1	n.a	48	15	154	Will not be detected
Vibrio parahaemolyticus	CP007006.1	n.a	n.a	3.7	n.a	Will not be detected
Vibrio cholerae	CP189261.1	308	12	n.a	n.a	Will not be detected
Vibrio cholerae	CP078724.1	308	12	n.a	n.a	Will not be detected
Vibrio cholerae	CP189268.1	308	12	n.a	n.a	Will not be detected

Vibrio cholerae	CP171689.1	308	12	n.a	n.a	Will not be detected
Vibrio cholerae	CP175806.1	77	12	n.a	n.a	Will not be detected
Vibrio cholerae	CP026531.1	77	12	232	616	Will not be detected
Vibrio cholerae	CP066156.1	77	3.0	232	154	Will not be detected
Vibrio cholerae	CP077198.1	308	3.0	n.a	n.a	Will not be detected
Vibrio cholerae	CP053815.1	n.a	n.a	924	n.a	Will not be detected
Vibrio cholerae	CP161854.1	n.a	12	n.a	616	Will not be detected

Figure S2. Electroactive Surface Area: A bar graph depicts the improvements in EASA at each stage of the fabrication process. A significant boost in conductivity and overall performance was observed after the introduction of the PANI layer.

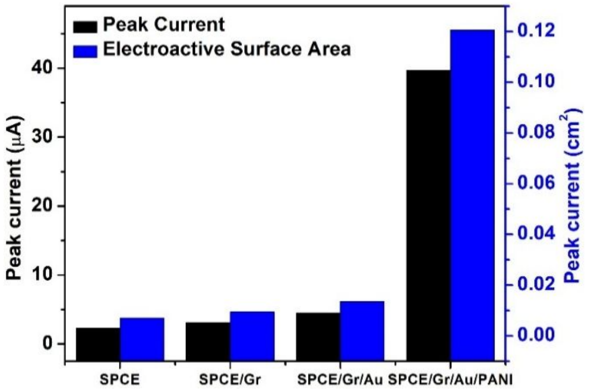

Figure S3. Functionalization: (a) Scanning electron microscopy (SEM) images of the -COOH functionalized graphene nanosheets synthesized, (b) FTIR spectra of aniline, SPCE/Gr (acid modified), and polyaniline (PANI) on SPCE/Gr/Au.

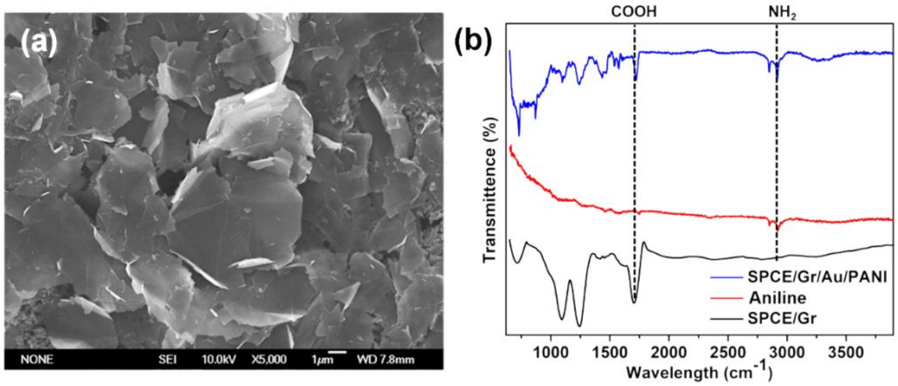

Figure S4. Au electrodeposition (LSV: -2.00 V to 0.00 V, w/o Ag/AgCl): SEM micrographs of the SPCE / Gr electrode after Au electrodeposition using LSV with a potential range of -2.00 V to 0.00 V in the absence of the Ag / AgCl reference electrode.

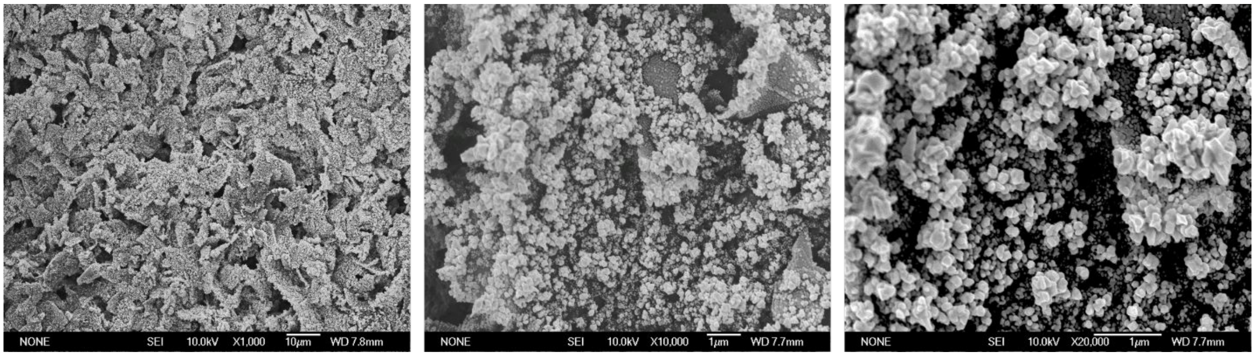

Figure S5. Au electrodeposition (LSV: -3.00 V to 0.00 V, w/o Ag/AgCl): SEM micrographs of the SPCE / Gr electrode after Au electrodeposition using LSV with the potential range of -3.00 V to 0.00 V in the absence of the Ag / AgCl reference electrode.

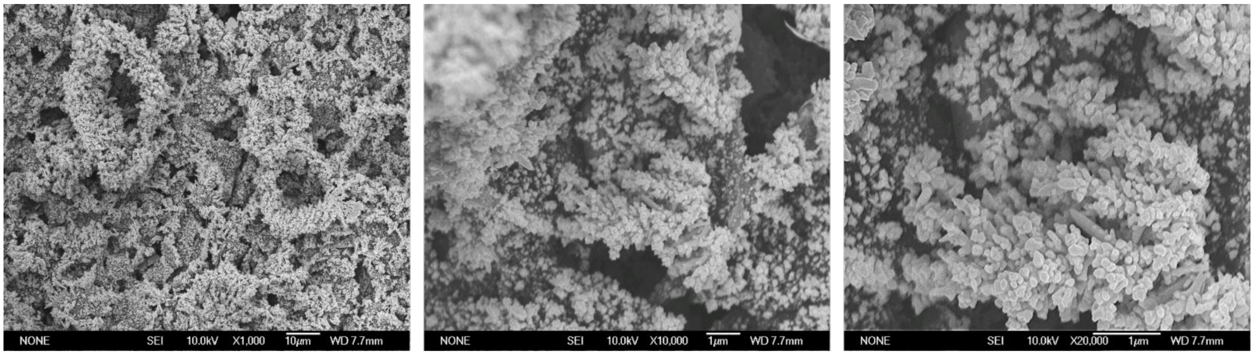

Figure S6. Au electrodeposition (LSV: -2.00 V to 0.00 V, w/ Ag/AgCl): SEM micrographs of the SPCE / Gr electrode after Au electrodeposition using LSV with a potential range of -2.00 V to 0.00 V in the presence of the Ag / AgCl reference electrode.

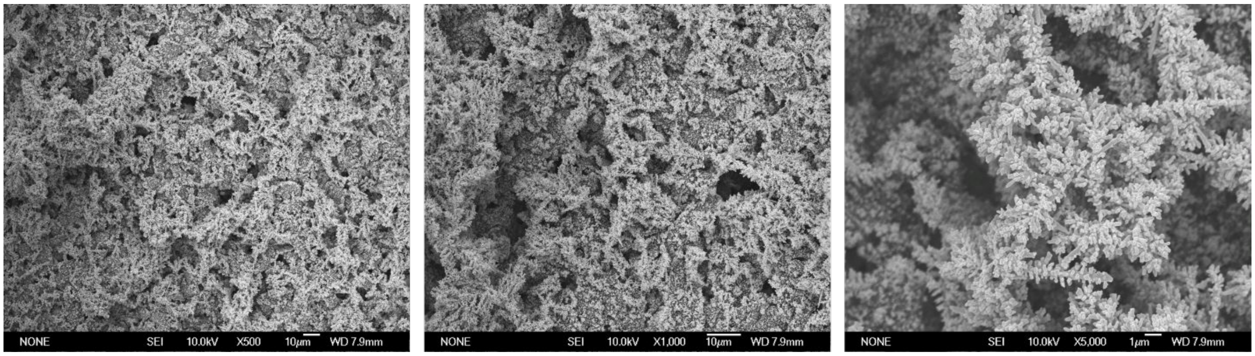

Figure S7. Au electrodeposition (LSV: -3.00 V to 0.00 V, w/ Ag/AgCl): SEM micrographs of the SPCE / Gr electrode after Au electrodeposition using LSV with the potential range of -3.00 V to 0.00 V in the presence of the Ag / AgCl reference electrode.

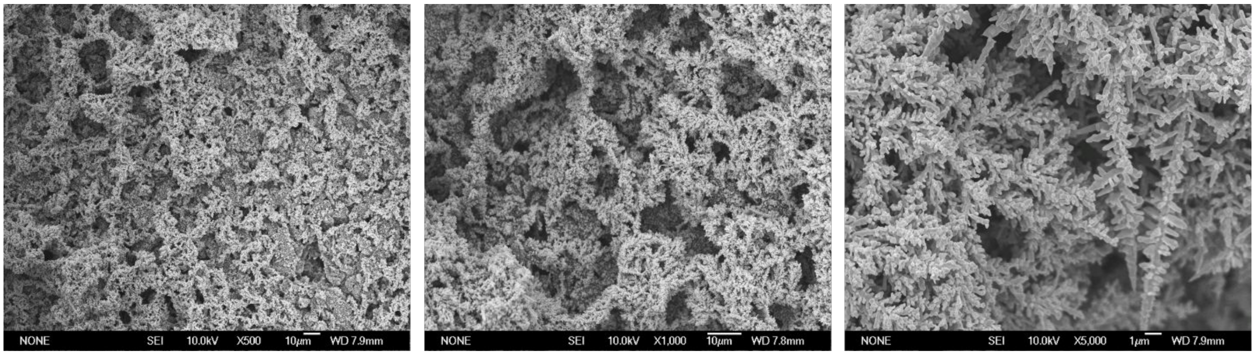

Figure S8. Au electrodeposition (CA: -5.00 Ma, 200 s, w/o Ag/AgCl): SEM micrographs of the SPCE / Gr electrode after Au electrodeposition using chronopotentiometry (CA) at -5.00 mA for 200 s in the absence of the Ag / AgCl reference electrode.

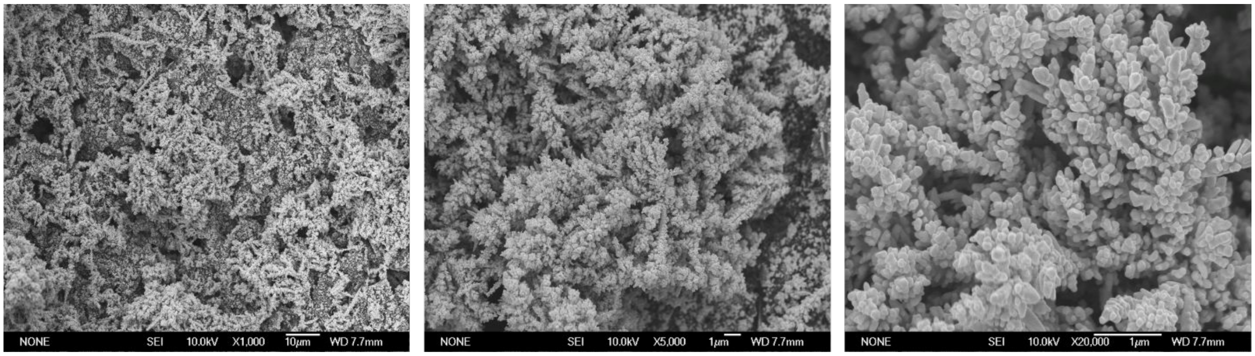

Figure S9. Au electrodeposition (CA: -5.00 Ma, 250 s, w/o Ag/AgCl): SEM micrographs of the SPCE / Gr electrode after Au electrodeposition using chronopotentiometry at -5.00 mA for 250 seconds in the absence of the Ag / AgCl reference electrode.

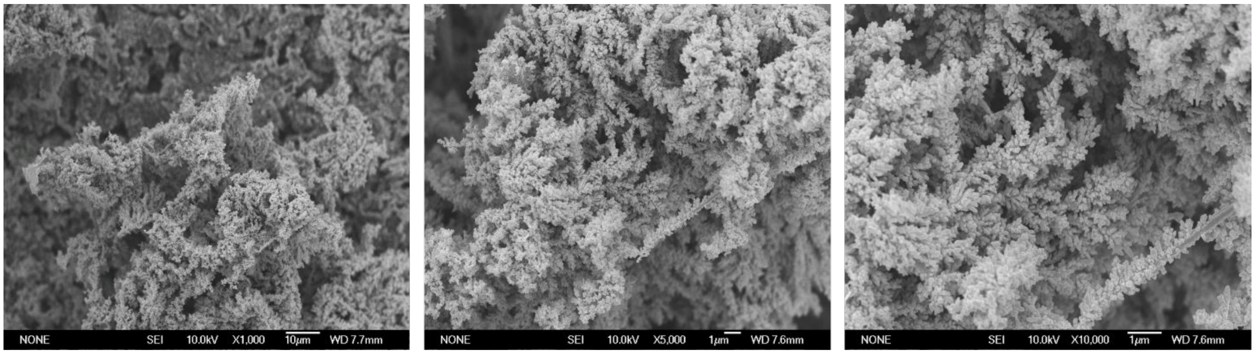

Figure S10. Au electrodeposition (CA: -5.00 Ma, 200 s, w/ Ag/AgCl): SEM micrographs of the SPCE / Gr electrode after Au electrodeposition using chronopotentiometry at -5.00 mA for 200 s in the presence of the Ag / AgCl reference electrode.

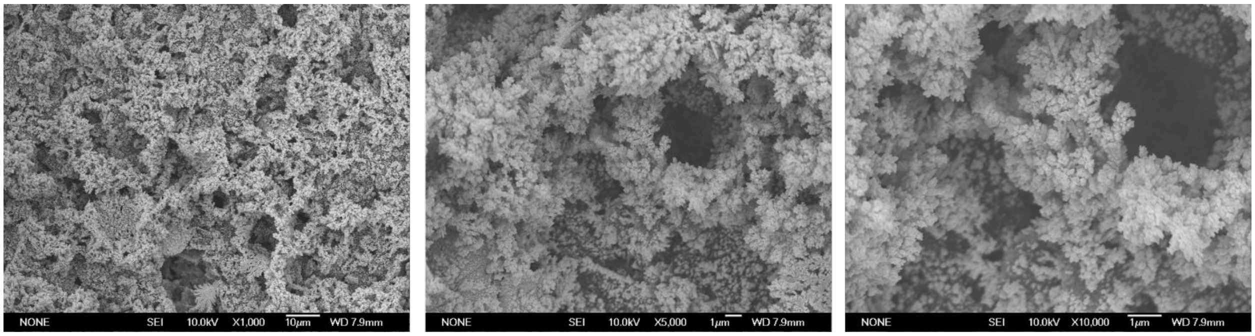

Figure S11. Au electrodeposition (CA: -5.00 Ma, 250 s, w/ Ag/AgCl): SEM micrographs of the SPCE / Gr electrode after Au electrodeposition using chronopotentiometry at -5.00 mA for 250 seconds in the presence of the Ag / AgCl reference electrode.

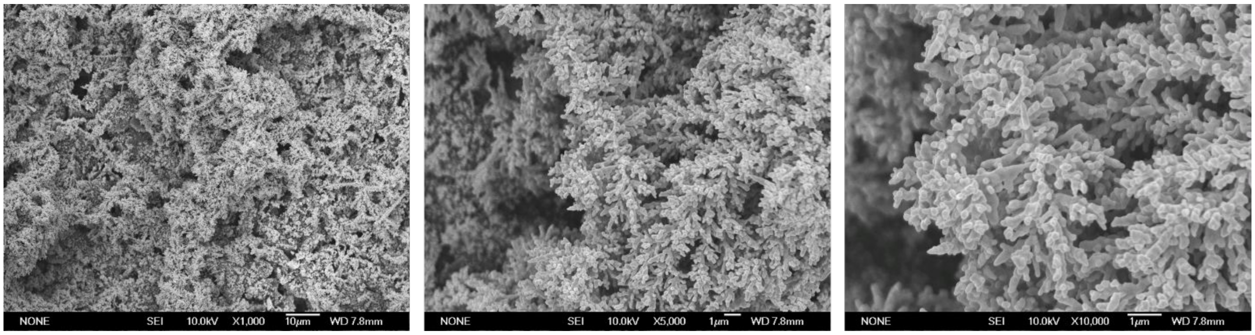

Figure S12. PANI electro polymerization w/o Ag/AgCl: SEM micrographs of the SPCE / Gr / Au electrode after aniline electro polymerization using cyclic voltammetry (potential range: -0.20 to -0.90 V, scan rate: 0.01 V/s) for 7 cycles in the absence of the Ag / AgCl reference electrode.

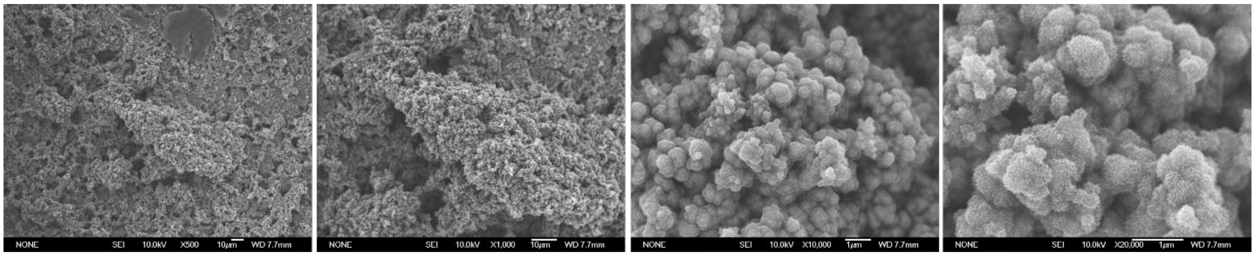

Figure S13. PANI electro polymerization w/ Ag/AgCl: SEM micrographs of the SPCE / Gr / Au electrode after electro polymerization of aniline using cyclic voltammetry (potential range: -0.20 V to -0.90 V, scan rate: 0.01 V/s) for 7 cycles in the presence of the Ag / AgCl reference electrode.

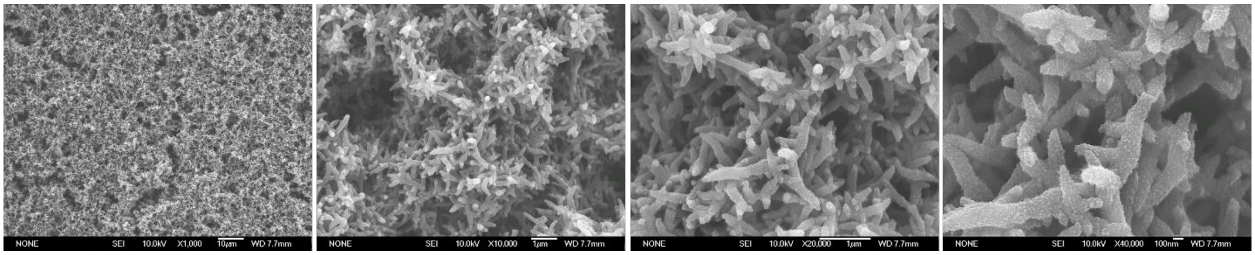

Table S5: HP quantification in spiked biological samples using the developed SPCE/Gr/Au/PAN biochip. The reliability and precision of the biochip was directly verified by comparing observed target DNA concentration(s) with the corresponding spiked (known) and clinically determined values.

**Sample Designation**	**HP DNA concentration (ng/µL)**			**Recovery (%)**
	**Spiked**	**Clinical**	**Biochip**	
Sample 1	10^2 Copies (52.00 ng/µL)	50.82	49.54	97.48
Sample 2	10^4 Copies (95.79 ng/µL)	95.19	94.38	98.53

Sample 3	10^6 Copies (620.71 ng/μL)	622.58	621.32	100.01
